# *NPR1* paralogs of Arabidopsis and their role in salicylic acid perception

**DOI:** 10.1371/journal.pone.0209835

**Published:** 2018-12-28

**Authors:** María José Castelló, Laura Medina-Puche, Julián Lamilla, Pablo Tornero

**Affiliations:** Instituto de Biología Molecular y Celular de Plantas, Universitat Politècnica de València -Consejo Superior de Investigaciones Científicas, Valencia, SPAIN; University of Maryland Baltimore County, UNITED STATES

## Abstract

Salicylic acid (SA) is responsible for certain plant defence responses and *N**ON EXPRESSER OF* *P**ATHOGENESIS* *R**ELATED 1* (*NPR1*) is the master regulator of SA perception. In *Arabidopsis thaliana* there are five paralogs of *NPR1*. In this work we tested the role of these paralogs in SA perception by generating combinations of mutants and transgenics. *NPR2* was the only paralog able to partially complement an *npr1* mutant. The null *npr2* reduces SA perception in combination with *npr1* or other paralogs. NPR2 and NPR1 interacted in all the conditions tested, and NPR2 also interacted with other SA-related proteins as NPR1 does. The remaining paralogs behaved differently in SA perception, depending on the genetic background, and the expression of some of the genes induced by SA in an *npr1* background was affected by the presence of the paralogs. NPR2 fits all the requirements of an SA receptor while the remaining paralogs also work as SA receptors with a strong hierarchy. According to the data presented here, the closer the gene is to *NPR1*, the more relevant its role in SA perception.

## Introduction

Plants use several pathways for their defence against pathogens. Broadly speaking, necrotrophic pathogens are dealt with by the methyl jasmonate pathway (MeJA, [1]), while biotrophic pathogens are resisted by means of the salicylic acid pathway (SA, [2]), with examples of positive and negative interactions between both pathways [3].

*N**ON EXPRESSER OF* *P**ATHOGENESIS* *R**ELATED 1* (*NPR1*) is an important SA perception gene. When mutants of *Arabidopsis thaliana* (Arabidopsis) were found that did not recognize SA or its analogues, the results were three alleles of *N**O* *R**ESPONSE TO* *B**TH 4* (NRB4, [4]), and more than fifty alleles of NPR1 [5–9]. NPR1 is a protein with several domains: a BTB/POZ (broad-complex, tramtrack, and bric-a-brac/poxvirus and zinc-finger) domain, and an ankyrin repeat domain. It also contains a nuclear localization sequence [10], and a transactivation domain in the C terminal [11].

NPR1 interactions have been described profusely. Among these interactors, there are some that negatively regulate its function, such as NIMIN1 (NIM1-INTERACTING 1, [12]), others that bind SA, such as βCAs (β CARBONIC ANHYDRASES, [13]), and others that bind to the promoters of defence genes, such as TGAs (members of the basic/leucine zipper-type transcription factors family, [14]). NPR1 binds SA and has been proposed as the SA receptor [15], although there alternative proteins proposed (see below). NPR1 is localized mainly in the cytoplasm in oligomeric form, and when SA is present, NPR1 is monomerized and concentrates in the nucleus [16]. NPR1 then activates TGAs and is regulated via degradation [17].

*NPR1* has five paralogs in Arabidopsis (*NPR2*, *NPR3*, *NPR4*, *B**LADE* *O**N* *P**ETIOLE 1* (*BOP1*), and *BOP2*). NPR3 and NPR4 (NPR3/4) have been shown to negatively regulate defences [18], perhaps through NPR1 [19] or independently [20].

BOP1 and BOP2 (BOP1/2) function redundantly, and the double mutant has a phenotype of ectopic blades along the petioles and some alterations in the flowers [21]. *bop1 bop2* behaves similarly to wild type (wt) plants for SA perception [22], but is impaired in MeJA perception in a specific phenotype related to defence against *Pseudomonas syringae pv*. *tomato DC3000 (Pto)* [23]. *NPR1* orthologs have been described in numerous species such as rice, apple, and tobacco ([24–26], respectively). No other paralogs or orthologs have been described that concentrate in the nucleus upon SA treatment like the Arabidopsis NPR1.

Plants with a deficient *npr1* allele such as *npr1-1* do not respond to SA or to its analogue benzothiadizole (BTH, [27]) in terms of defence or plant growth [22]. However, SA is able to induce the expression of some genes in an *npr1* background [28]. Plants with NPR1 null alleles have a small but measurable response to SA in terms of defence and plant growth [9], which suggests that there may be other SA receptors.

We reasoned that the most likely candidates were the *NPR1* paralogs (henceforth abbreviated to *NPRs*), an idea that had already been suggested in a previous study [9]. NPR3/4 have been proposed to act as SA receptors by regulating the NPR1 protein [19], in disagreement with NPR1 being the SA receptor [15]. In this paper we searched for the *NPRs’* role in SA perception using several approaches and found that the *NPRs*, especially *NPR2*, do play a role in SA perception.

## Material and methods

### Plant growth and inoculation

Arabidopsis (*Arabidopsis thaliana* (L.) Heynh.) and *Nicotiana benthamiana* were sown and grown as described [22], in controlled environment rooms with days of 8 h at 21°C, 150 μmol m^-2^ s^-1^ of light intensity and nights of 16 h at 19°C. The treatments, inoculations, and sampling started 30 minutes after the initiation of the artificial day to ensure reproducibility. The following genotypes were used: *npr1-1* [7], *npr3 npr4* [18], *bop1 bop2* [29], *npr2* (N622643 from NASC, www.arabidopsis.info). *35S*:*GFP-NPR1* in *npr1-70* [23], *35S*:*GFP-NPR1* in *npr1-1* [23], *35S*:*NPR1-GFP* in *npr1-1* [30], *35S*:*NPR1* [31], and *35S*:*GFP-NRB4* [4].

*Pseudomonas syringae pv*. *tomato* DC3000 (*Pto*) was grown, inoculated and measured as described [32]. Briefly, 14-day old plants were inoculated by spray with *Pto* at 5*10^7^ colony forming units (cfu) per mL with 0.02% Silwet L-77 (Crompton Europe Ltd, Evesham, UK). Three days later the amount of cfu per plant was quantified and represented on a logarithmic scale. In the eds-like experiment, twelve seven-week-old plants were hand inoculated with a needleless syringe containing *Pto* at 5*10^4^ cfu/mL. Three leaves of each plant were completely infiltrated, and three days later the inoculated leaves were collected, weighed, and the amount of bacteria measured. For all the experiments, at least three independent treatments were performed (three independent sets of plants sown and treated on different dates). The statistical analyses were performed with Excel 2007 (Microsoft, Redmond, WA, USA), and R [33].

### Chemical treatments

Primers and chemical products were purchased from SIGMA (St. Louis, MO, USA) unless otherwise stated. Benzothiadiazole (BTH, CGA 245704), in the form of a commercial product (Bion 50 WG, a gift from Syngenta Agro S.A., Madrid, Spain) was prepared in water for each treatment and applied with a household sprayer. The response to BTH in terms of fresh weight was done as reported [22]. In short, plants were treated with mock or 350 μM BTH four times over three weeks, after which the fresh weight of the plants was recorded and expressed as the ratio between BTH and mock treated plants. SA (in the form of sodium salicylate), and 4-hydroxybenzoic acid (HBA) were applied at 1 mM unless otherwise is stated.

### SA in plates and *in planta*

Arabidopsis seeds were surface-sterilized for 10 min in ethanol and for 10 min in 1% formaldehyde and then washed five times with distilled water before distributing the seeds on agar plates. The medium contained 0.5x Murashige and Skoog salts (Duchefa BV, Haarlem, the Netherlands), 0.6% (w/v) Phyto Agar (Duchefa), 2% (w/v) sucrose, with 0, 200 or 300 μM SA (final concentration, in the form of sodium salicylate). The results were evaluated 7 days after transfer to growing conditions.

### Yeast experiments

The cDNAs of the *NPR1* paralogs were cloned in pDONR222 (Invitrogen, Barcelona, Spain) from RT-PCR, and then transferred to pDEST22 and pDEST32 (Invitrogen) for expression in yeast. Additionally, the pARC352 vector [34] was used for the triple interaction. Yeast n-hybrid analyses were carried out as described in [35]. Briefly, yeast was transformed with two cDNAs, one in pDEST22 and one in pDEST32. If the yeast was able to grow on a plate with no histidine, it was considered as an interaction. Since some proteins have a small background (autoactivation [4], as NPR2 (this work) or NRB4 [4]), the plates were supplemented with 5 mM or less of 3-Amino-1,2,4-triazole (3AT, an inhibitor of histidine biosynthesis) when indicated. Also, the yeast plates were supplemented with 100 μM SA (in the form of sodium salicylate), or 100 μM HBA, as specified in the figures.

### Expression *in planta* and microscopy

The cDNAs of the *NPR1* paralogs were transferred to pMDC43 [36] for expression of GFP fused proteins *in planta*. The plasmid pMDC-MBP was also used (a gift from Drs. Carrasco and Vera, IBMCP) for expression of Maltose Binding Protein fused proteins. For the BiFC experiments, the cDNAs were cloned in pYFC43 and pNFC43 [37]. *N*. *benthamiana* leaf tissue was mounted in water under a coverslip 4 days after infiltration with *Agrobacterium tumefaciens* containing the constructs. When indicated, plants were treated with 1 mM SA by spray and collected or visualized one day later. The Arabidopsis transgenic plants were three weeks old when photographed. A Leica TCS SL confocal laser scanning microscope (Leica, Heidelberg, Germany) using an HCX PL APO CS 40X/1.25 water objective was used to study the subcellular localization of the fluorescence-tagged proteins. Green fluorescent protein was visualized by 488-nm excitation with an Ar laser, and its emissions were examined with a band-pass filter for 500 to 530 nm.

### RT-qPCR

Total RNA was extracted with RNAzole RT (SIGMA), following the manufacturer’s instructions. cDNA was synthesized with RevertAid First Strand cDNA Synthesis Kit (Fermentas, Madrid, Spain), and the quantitative PCR performed with LuminoCt Sybr Green qPCR Ready Mix (SIGMA) in a 7500 Fast RT-PCR Systems machine (Applied Biosystems, Madrid, Spain), following the manufacturer’s instructions. Three biological replicates were performed for each measurement. The obtained values were referred to the geometric average of three reference genes (At3G18780, At1G49240, and At5G60390), as described [38], and normalized, the value of Col-0 in mock (or HBA when indicated) being equal to one. The list of primers used is provided in S1 Table.

### Immunoblots and co-sedimentation assays

Extracts and co-sedimentation assays were done essentially as published [13] by using an amylose resin (New England Biolabs, Schwalbach, Germany). For immunoblots analysis the following antibodies were used: rabbit polyclonal anti-GFP N-terminal antibody (SIGMA), rabbit polyclonal anti-maltose binding protein antibody (Abcam, Cambridge, UK), and rabbit polyclonal anti-PR1 from tobacco [39] kindly provided by S. Kauffmann (IBMP, Strasbourg, France). The secondary antibody was anti-rabbit IgG-peroxidase conjugate (SIGMA) and we used Amersham ECL Plus Western Blotting detection reagents (GE HealthCare, Little Chalfont, UK) detected with a LA-3000 Luminescent Image Analyzer (Fujifilm Life Science, Stamford, CT, USA).

### Protein expression in *E*. *coli* and SA binding activity

*NPR1* paralogs cDNAs were cloned into pHMGWA and expressed as described [40]; [13]. The proteins were purified by amylose affinity chromatography (New England Biolabs) and eluted with maltose. The SA binding activity assay was done as described [41]; [13]. In short, proteins were incubated 50 μM 4-AzSA (Santa Cruz Biotechnology, Dallas, TX, USA), followed by UV irradiation. Reaction mixtures were subjected to SDS–PAGE, and 4-AzSA crosslinked proteins were detected by immunoblot analyses using a sheep anti-SA antibody (Fitzgerald Industries International, Acton, MA, USA).

### Accession numbers

Sequence data from this article can be found in the Arabidopsis Genome Initiative or GenBank/EMBL databases under the following accession numbers: NRB4, At1g15780; NPR1, At1g64280; NPR2, At4g26120; NPR3, At5g45110; NPR4, At4g19660; BOP1, At3g57130; BOP2, At2g41370; αDOX1, At3g01420; GRX480, At1g28480; OPR1, At1g76680; PR1, At2g14610; ANAC102, At5g63790; UGT1, At1g05560; NIMIN1, At1g02450; TGA2, At5g06950; TGA5, At5g06960; TGA6, At3g12250; TGA7, At1g77920; CA1, At3g01500; CA2, At5g14740; CA3, At1g23730; CA4, At1g70410.

## Results

### NPR1 like function in *NPR1* paralogs

Our first approach was to transiently express the *NPRs* of Arabidopsis in *Nicotiana benthamiana* under the control of the promoter *35S*. In order to check if there was any NPR1-like activity, our readout was the expression of the PR1 protein [42]. S1a Fig shows the immunodetection of PR1 after the transient expression of the Green Fluorescence Protein (GFP) fused to the NPRs, while the bottom shows the level of the GFP-NPRs as a control. The same experiment was repeated (S1b Fig) after an SA spray one day prior to the sampling. Without SA, NPR2 produced a stronger signal than NPR1, although the rest of the paralogs also induced PR1 expression to some degree. The expression of NPR1 along with exogenous SA produced an increase in PR1 with respect to the empty vector control. S2 Fig shows the images of the GFP experiment checked by confocal microscopy. NPR1 has been observed to concentrate in the nucleus with SA treatment [30]. The rest of the paralogs did not change their localization with SA, NPR2, NPR3, and NPR4 being in the nucleus, and BOP1 and BOP2 in the cytosol and nucleus.

We then transformed Arabidopsis *npr1-1* plants [7] with all the *35S*:*GFP-NPRs*. The plants were tested in T2 by applying SA, and then by inoculating with *Pto*. Out of 33 lines transformed with *35S*:*GFP-NPR1*, 5 lines showed a reduction of the symptoms caused by *Pto* (i.e. complementation), with a clearly dominant segregation. When lines transformed with *35S*:*GFP-NPR2* were tested, 12 of 33 lines showed complementation. There was no complementation when lines transformed with *35S*:*GFP* fused to the empty vector, *NPR3*, *NPR4*, *BOP1*, or *BOP2* were tested (33 lines for each).

**Fig 1:**
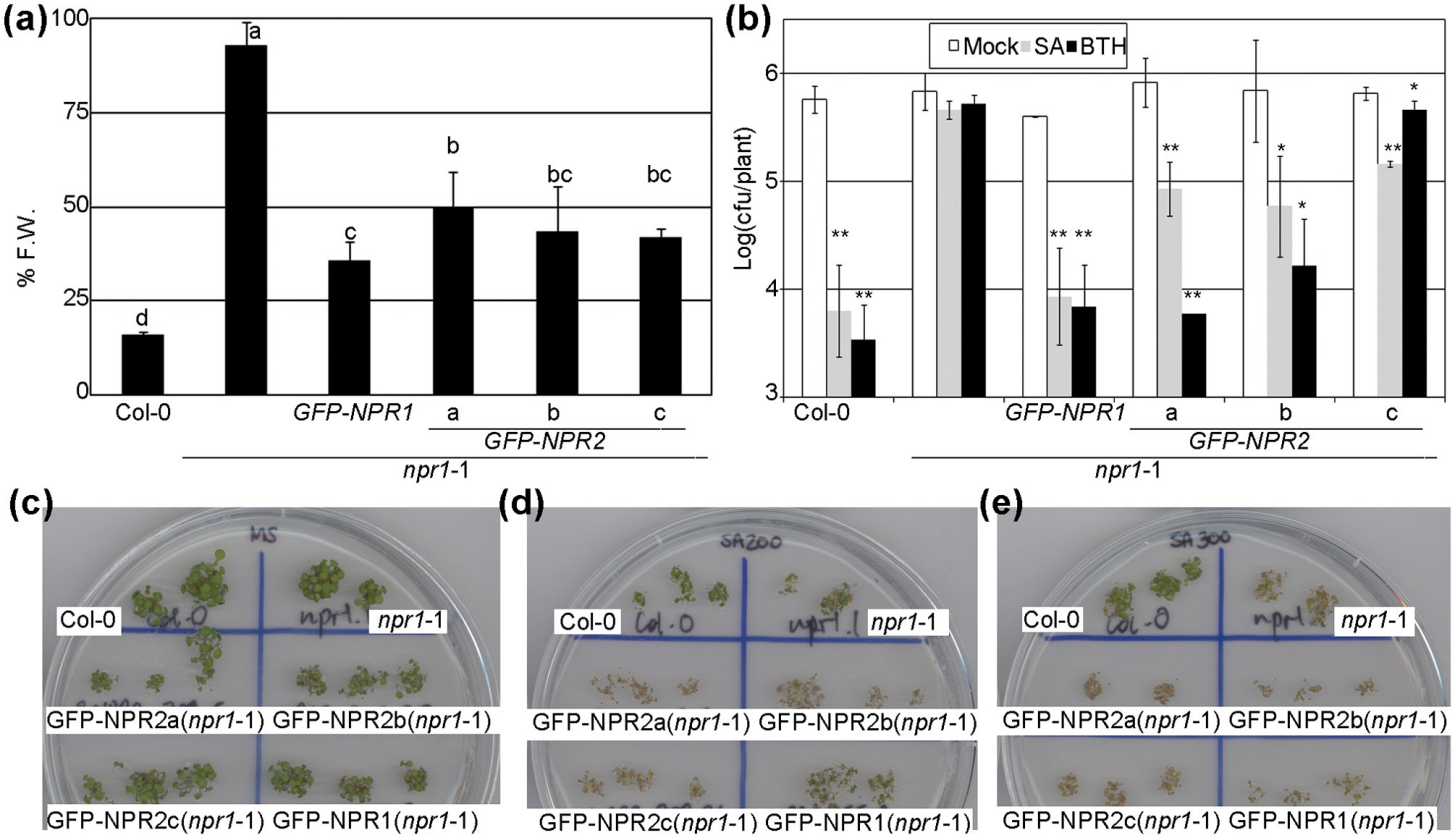
*NPR2* complements *npr1-1* in Arabidopsis. (a) Homozygous transgenic plants *35S*:*GFP-NPR2* of Arabidopsis in an *npr1-1* background were tested for their response to benzothiadizole (BTH, an analogue of SA), along with control genotypes. The response to BTH was measured as weight, and plants were treated with either mock or 350 μM BTH. The ratio is expressed as percentage of fresh weight (%FW). The letters above the bars indicate different homogeneous groups with statistically significant differences (Fisher’s LSD Test, P < 0.05). In all the figures that give numerical information, the data represent the average, with the error bars plotting the standard deviation. All the experiments were repeated at least three times with similar results. (b) The indicated genotypes (14-day-old plants) were treated with either 1 mM SA, 350 μM BTH or a mock solution. One day later the plants were inoculated with *Pseudomonas syringae* pv. *tomato* isolate DC3000 (*Pto*) at 5*10^7^ colony forming units (cfu) per mL. Three days after inoculation, the growth of *Pto* was evaluated as the Logarithm of cfu per plant. Asterisks indicate statistically significant differences from the mock treatment in each genotype (P < 0.05 one asterisk, P < 0.01 two) using the Student’s t test (one tail). (c) The indicated genotypes were grown on MS plates, and the picture was taken at day 7 after germination. (d) The same experiment as in (a), with 200 μM SA. (e) The same experiment as in (a), with 300 μM SA.

Three independent *35S*:*GFP-NPR2* lines that complemented the *npr1-1* background were randomly selected and taken to homozygosis. Fig 1a shows the response of these three lines to BTH in terms of plant weight [22]. We included a *35S*:*GFP-NPR1* in *npr1-1* line generated with the same vector as a control. The three *35S*:*GFP-NPR2* lines partially complemented the *npr1-1* mutant, not very different from the control *35S*:*GFP-NPR1*. The second phenotype tested was the induction of defence against a pathogen; the same genotypes were treated with water, SA, and BTH, and one day later were inoculated with *Pto* (Fig 1b). Clearly the *35S*:*GFP-NPR2* lines, while responding to SA, were intermediate in this phenotype. The third phenotype tested was the growth of the plants on a plate with SA. In this condition, *npr1-1* plants do not develop true leaves, and the cotyledons are bleached [7]. Fig 1c shows that all the genotypes tested grew normally on plates without SA, while in 200 μM SA (Fig 1d) and 300 μM SA (Fig 1e), *npr1-1* was affected. The *35S*:*GFP-NPR2* lines did not complement the *npr1-1* mutation, although the control *35S*:*GFP-NPR1* was not completely wt. S3 Fig shows the images of the transgenic lines used, visualized by confocal microscopy after SA or mock treatment. We also included two NPR1 additional controls for reference (S3c–S3f Fig). As with *N*. *benthamiana*, NPR2 was localized in the nucleus whether or not SA had been added. The third line, *35S*:*GFP-NPR2c* had a level of expression below our detection limit.

### Phenotype of knock out NPRs

**Fig 2:**
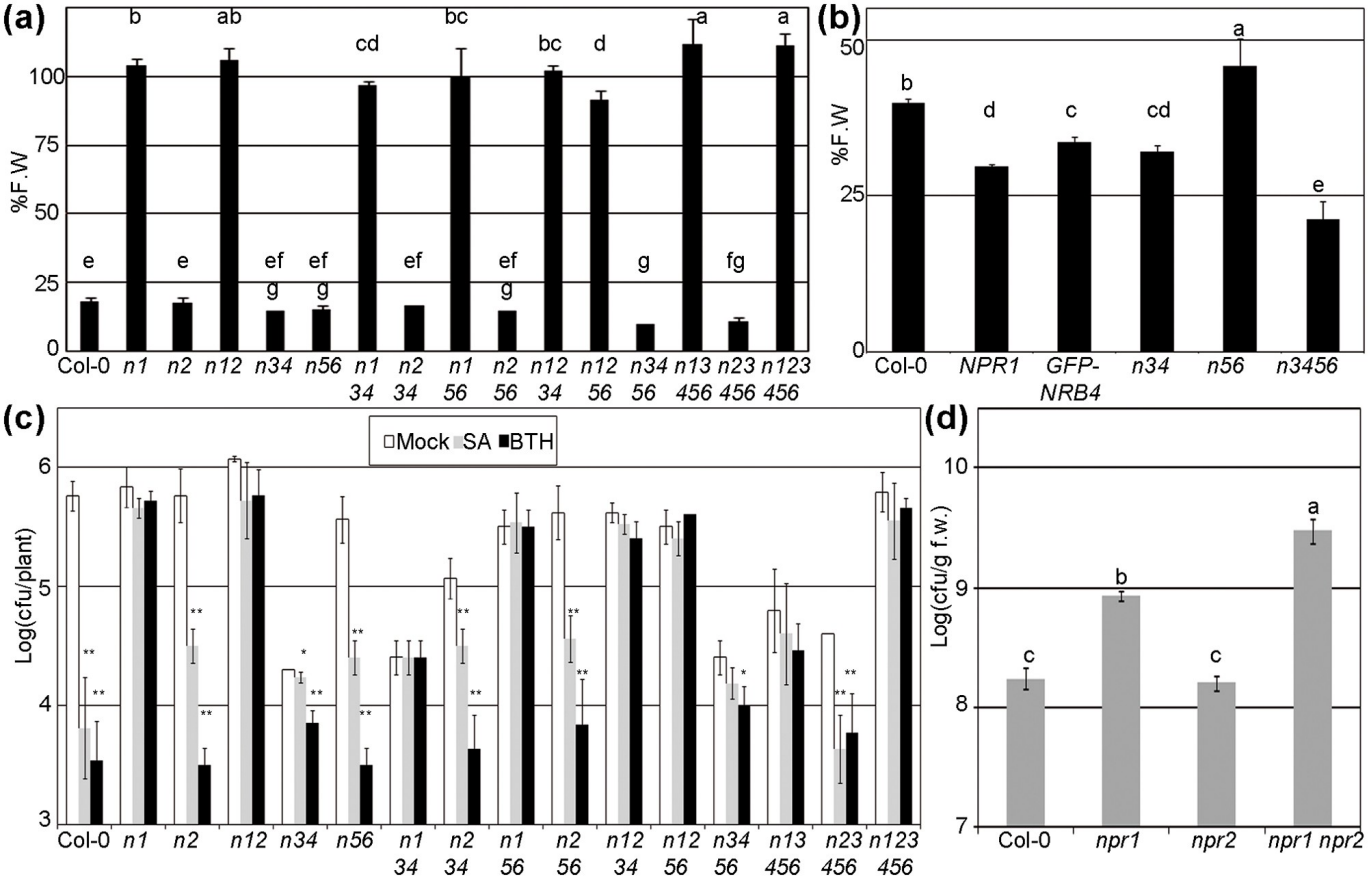
Phenotypes of the *NPR1* paralogs knockouts. (a) SA perception of combinations of KOs, measured by fresh weight after BTH treatment, as in Fig 1a. The abbreviations used are: *n1 = npr1-1; n2 = npr2; n3 = npr3; n4 = npr4; n5 = bop2; n6 = bop1*. The combinations of mutants are indicated by the letter “n” and the corresponding numbers, e.g. *n1234* correspond to the quadruple *npr1-1 npr2 npr3 npr4*. (b) Enhanced SA perception of the quadruple *npr3 npr4 bop1 bop2*. Plants were treated and measured as in (a), but with a ten-fold reduction in the BTH, to visualize increased sensitivity to SA. *35S*:*NPR1* and *35S*:*GFP-NRB4* are included as controls of enhanced perception ([31] and [4] respectively). (c) SA perception, measured as pathogen growth as in Fig 1b. (d) eds-like phenotype. Seven-week-old plants were hand infiltrated with *Pto* at 5*10^4^ cfu/mL. Three days after inoculation, the growth of *Pto* was evaluated as the Logarithm of cfus per g of fresh weight.

We then constructed the sextuple mutant that lacks all the NPRs, and several combinations of KOs in the paralogs. Fig 2a shows these KO combinations upon BTH treatment. Adding *npr2* to *npr1-1* did not change anything, but surprisingly, the sextuple mutant grew even better than *npr1-1*. *npr3 npr4* and *bop1 bop2* both responded as wt to BTH, as reported [22], but the combination *npr3 npr4 bop1 bop2* had an even stronger response than their parents (Fig 2a). The experiment was repeated with 35 μM BTH (ten times less). Fig 2b shows that again *npr3 npr4 bop1 bop2* had a stronger response than their parents, in fact even stronger than the transgenic lines that overexpressed *NPR1* or *NRB4* [4].

Another phenotype tested was the growth of *Pto* after mock, SA, or BTH treatment (Fig 2c). In these experiments, *npr2* plants behaved as wt and *npr3 npr4* had a constitutive expression of defence [18]. But the combination *npr2 npr3 npr4* produced plants closer to wt. A similar effect happened in *npr1-1 npr3 npr4* and *npr1-1 npr3 npr4 bop1 bop2*. These lines did not respond to SA or BTH, but there was a certain level of resistance already in the mock treatment. However, when *npr2* was introduced (*npr1-1 npr2 npr3 npr4*, and *npr1-1 npr2 npr3 npr4 bop1 bop2*, respectively) there was less resistance and they were closer to *npr1-1* alone.

The genotype *npr1-1 npr2* was more susceptible than *npr1-1* alone by a narrow margin. This difference can be seen more clearly with the eds-like phenotype (Fig 2d). In this experiment, adult plants are inoculated by hand with a small amount of *Pto* [6], and the result is that *npr2* in an *npr1-1* background produced plants more susceptible to *Pto*.

There were no significant phenotypes of these genotypes on plates with SA (S4 Fig), nor was there any significant difference between *bop1 bop2* and the sextuple mutant, regarding the “blade-on-petiole” phenotype (S5 Fig).

### Gene expression in mutant combinations

**Fig 3:**
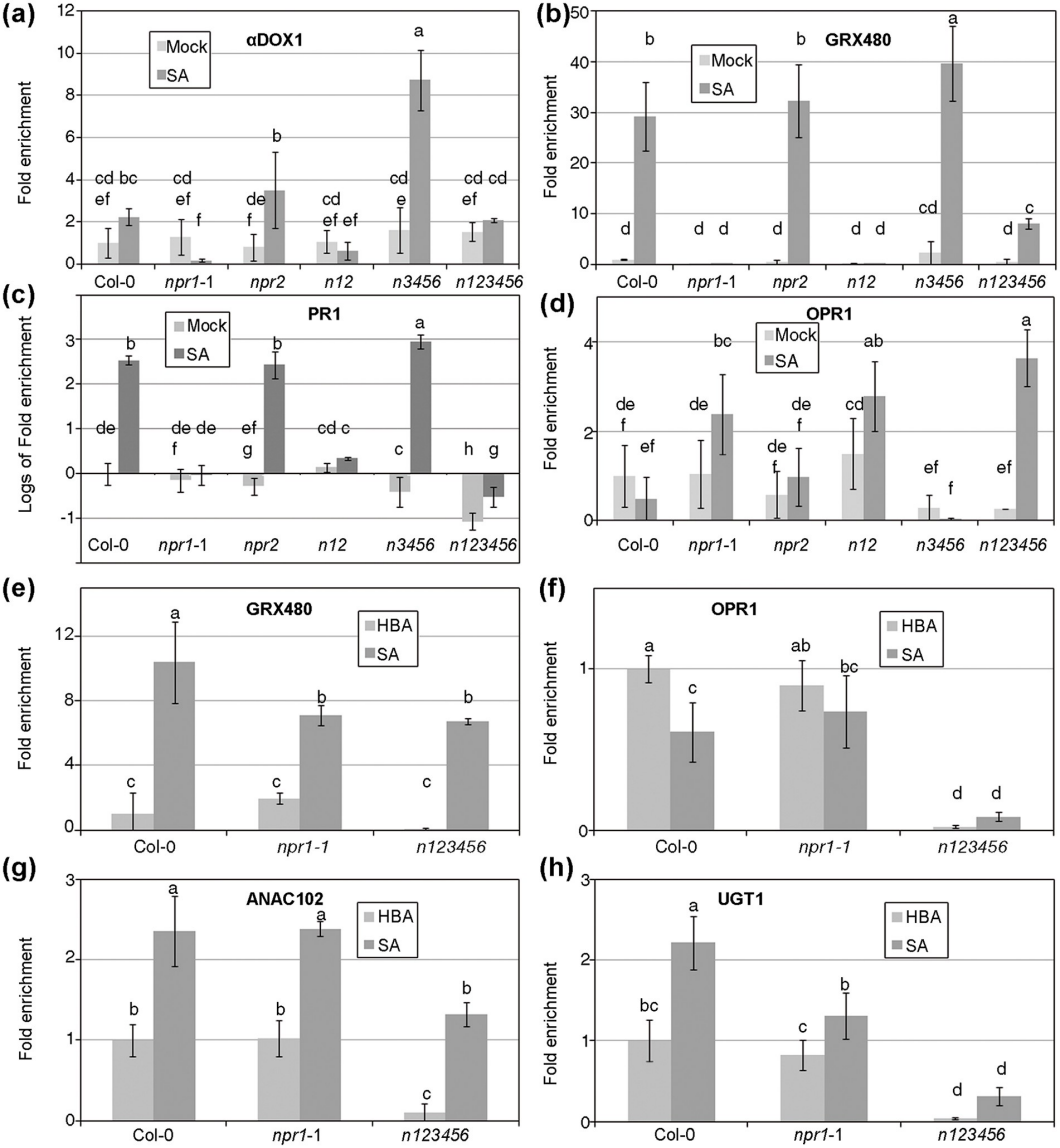
Gene expression depending on *NPR1* paralogs. RNA was extracted from two week old plants of the indicated genotypes. In the (a) to (d) panels, the samples were taken one day after treatments of mock or 1 mM SA, while in the (e) to (h) panels, the samples were taken 2.5 h after treatment of 1 mM 4-hydroxybenzoic (HBA, a isomer of SA with no biological activity) or 1 mM SA. (a) Fold enrichment of *α -Dioxygenase 1*, one day after treatment. (b) Fold enrichment of *Glutaredoxin 480*, one day after treatment. (c) Fold enrichment of *PR1* one day after treatment, in logarithmic scale. (d) Fold enrichment of *12-Oxophytodienoate reductase 11*, one day after treatment. (e) Fold enrichment of *Glutaredoxin 480*, 2.5 h after treatment. (f) Fold enrichment of *12-Oxophytodienoate reductase 11*, 2.5 h after treatment. (g) Fold enrichment of *NAC domain containing protein 102*, 2.5 h after treatment. (h) Fold enrichment of *UDP-glucose transferase 1*, 2.5 h after treatment. The transcript levels of the indicated genes were measured by RT-qPCR, and the levels of expression are normalized to three reference genes and to the level of Col-0 in mock or HBA treatment.

One phenotype frequently measured in SA perception is the expression of well-known marker genes. In our case, we choose genes induced by SA in wt, especially those also induced in an *npr1* mutant (or SA dependent, NPR1 independent (*SdNi*), [43];[44]). Fig 3a shows the expression of *α dioxygenase1* (*αDOX1*, [45]) in several genotypes treated with mock or SA, one day before the sampling. In these conditions, the *npr3 npr4 bop1 bop2* plants expressed much more *αDOX1* upon SA treatment than the wt. A similar effect was detected in the case of one *glutaredoxin* (*GRX480* [46], Fig 3b). The third gene studied was *PR1*, a gene strictly dependent on SA and NPR1, included as a control (Fig 3c). The results are expressed on a logarithmic scale, since the range expands by several orders of magnitude. Again, the induction of this gene in *npr3 npr4 bop1 bop2* was stronger than the wt. The fourth gene in this set was *12-Oxophytodienoate Reductase 1* (*OPR1* [47]). Fig 3d shows that this gene was repressed in wt and in *npr3 npr4 bop1 bop2*. *OPR1* was also induced in *npr1-1*, and in all the genotypes that contained this mutation. Surprisingly, the induction of *OPR1* in the sextuple mutant is stronger than in *npr1-1* alone. In fact, the behaviour of the sextuple mutant in these four genes was quite different from *npr1-1* or *npr1-1 npr2*, especially in the presence of SA. Since the *SdNi* genes have been described at very short times after SA treatment, we carried out a new experiment, with three genotypes and samples taken 2.5 h after SA spray. It is theoretically possible that the genes were induced by the chemical characteristics of SA, regardless of the specific recognition of SA in defence. To check this point, we used 4-hydroxybenzoic acid (HBA, an isomer of SA with no biological activity) as a mock control. We repeated two genes from the previous experiment, *GRX480* (Fig 3e) and *OPR1* (Fig 3f), and the results were quite different. For *GRX480* with SA, the sextuple phenocopies *npr1-1*, while for *OPR1* with SA the expression of the sextuple is quite different from *npr1-1*. Two more genes described as *SdNi*, *Nac Domain Containing Protein 102* (*ANAC102* [44]), and *Udp-Glucose Transferase 1* (*UGT1* [48]), were tested in our conditions (Fig 3g and 3h, respectively). In both cases, the levels of expression in the sextuple were lower than *npr1-1*, as with *OPR1*, indicating that part of their regulation is independent of *NPR1*, but dependent on the rest of *NPRs*. In these four genes at 2.5 h (Fig 3e–3h), the levels of expression for HBA treated plants in the sextuple were quite low. Clearly, HBA did not induce these genes in a similar fashion to SA, so the *SdNi* induction is quite specific to SA. Our data seem to show a paradox. *npr3 npr4 bop1 bop2* have an enhanced response to SA, but the same mutations introduced in an *npr1-1 npr2* background enhanced the phenotypes of insensibility to SA, in five of the genes and conditions tested. And in two genes and conditions the sextuple is closer to the wt than to *npr1-1 npr2* (Fig 3a and 3b).

### Protein interactions among the NPRs

**Fig 4:**
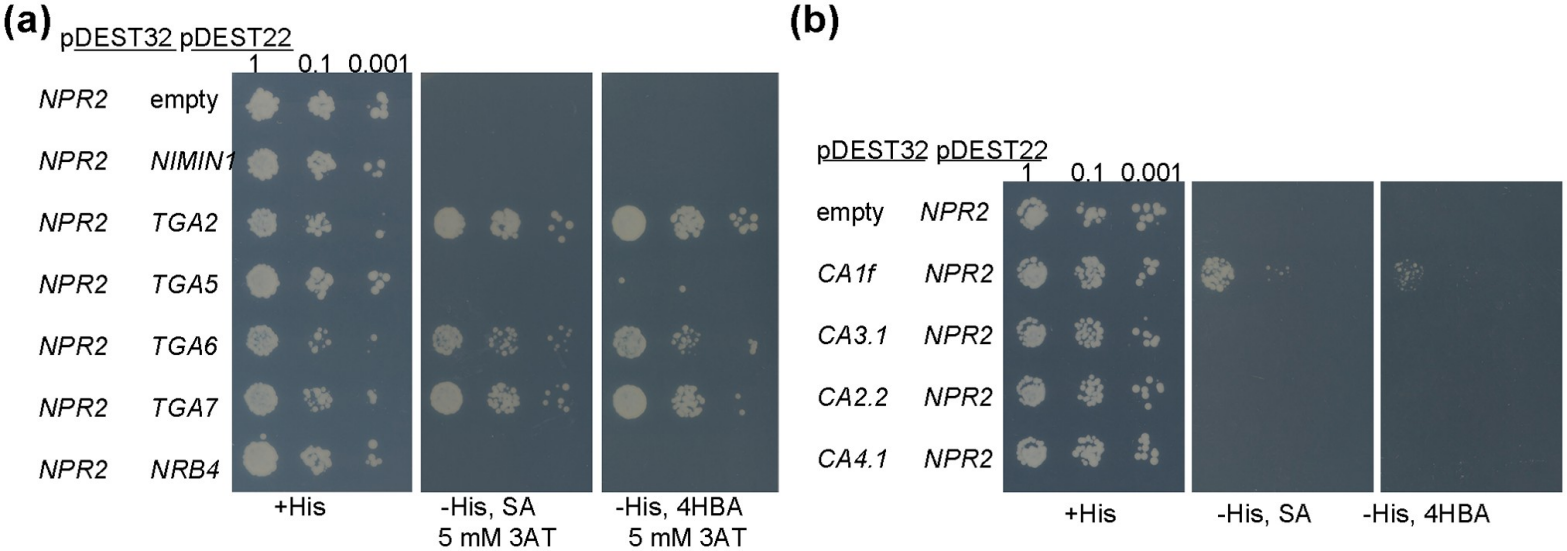
NPR2 interactions. (a) Interactions of NPR2 with proteins that interact with NPR1. Yeast transformed with the indicated plasmids and inserts were grown on three different sets of plates, by depositing a 0.5 μL drop of OD600s 1, 0.1, and 0.01 (indicated in the top of the first plate). The first plate contained minimal media supplemented with histidine (+His). The second had the same minimal media with no histidine (-His), 100 μM SA, and 5 mM 3-Amino-1,2,4-triazole (3AT), while the third plate is -His, 100 μM HBA, and 5 mM 3AT. (b) Interactions of NPR2 with β carbonic anhydrases (βCAs), as in (a) but without 3AT.

NPR2 has a function in SA perception, but does NPR2 work like NPR1? We tested whether NPR2 interacted with some of the proteins described as NPR1 interactors in the yeast two-hybrid system (Fig 4a and 4b). NPR2 did not interact with the repressor NIMIN1, but it did interact with some members of TGAs. While NPR2 interacted in our conditions with TGA2, TGA6, and TGA7 (Fig 4a), NPR1 interacted with all four tested (the same constructs were used in [4]). Fig 4b shows that NPR2 only interacts with one of the four βCAs proteins tested, whereas NPR1 was able to interact with all of them [13]. In all the cases tested, there was interaction with no SA in the media. Only in the case of βCA1f, SA increased the interaction with NPR2, while with NPR1 it was necessary [13].

**Fig 5:**
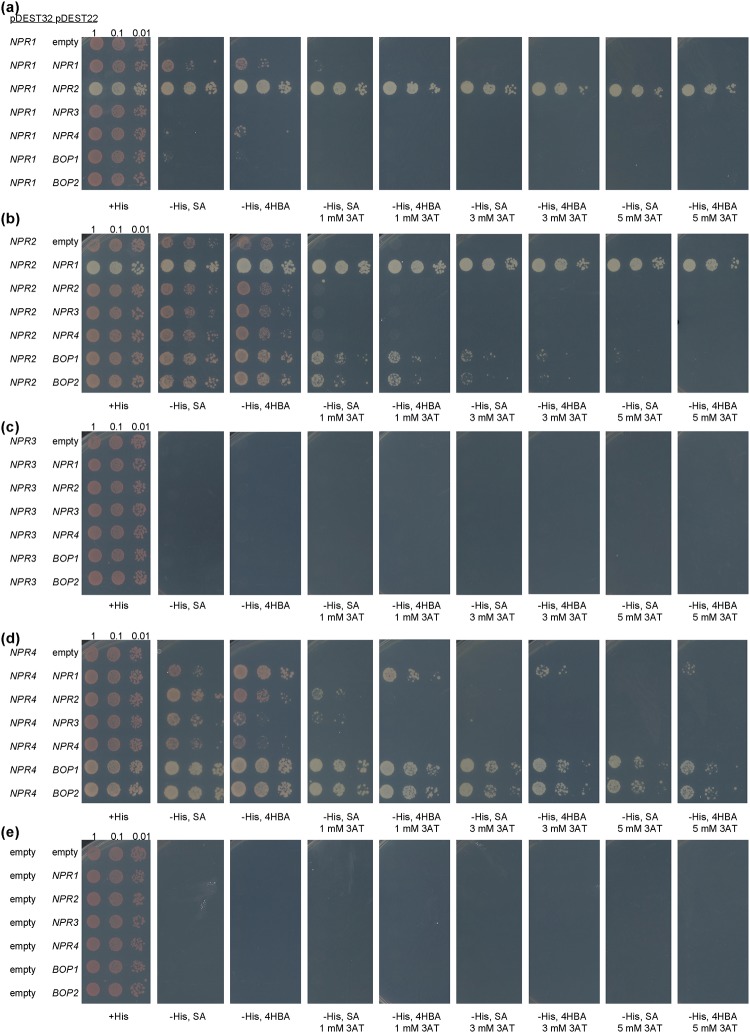
NPR1 paralogs in yeast two hybrid. (a) Interactions of NPR1 with the paralogs. (b) Interactions of NPR2 with the paralogs. (c) Interactions of NPR3 with the paralogs. (d) Interactions of NPR4 with the paralogs. (e) Controls of empty plasmids.

NPR1 has also been found to interact with NPR2, NPR3, and NPR4 [19]. In our conditions and among the paralogs, NPR1 interacted strongly in yeast with NPR2 (Fig 5a), with SA improving the interaction. When no 3AT was present, NPR1 also interacted with itself and with NPR4 in the absence of SA (Fig 5a). When the vectors were switched, NPR2 interacted strongly with NPR1 and less so with the BOPs (Fig 5b). The same interaction between NPR4 and NPR1 depending of the absence of SA could be found when the vectors were switched. Thus, NPR4 interacted with NPR1 in the presence of 0 to 5 mM 3AT. In the presence of SA (Fig 5d), the interaction between NPR4 and NPR1 is disrupted consistent with the data published [19]. Both NPR2 and NPR3 interacted with NPR4 in the presence of SA (Fig 5d), while NPR4 interacted with the BOPs regardless of the presence of SA (Fig 5d).

As mentioned, the interaction between NPR1 and βCA1f is dependent on SA. However, if NRB4 (an interactor of βCA1f) is introduced, the interaction is independent of SA [13]. We tested if any of the NPRs could have a similar effect, and only NPR2 had some effect if we grew the yeast long enough to see the colonies in the control (S6a Fig). Perhaps the interaction of NPR1 or NPR2 with NPR3 and NPR4 would be improved with NPR3 and NPR4 both present. We tried several combinations in yeast, but did not detect any interaction (S6b Fig).

**Fig 6:**
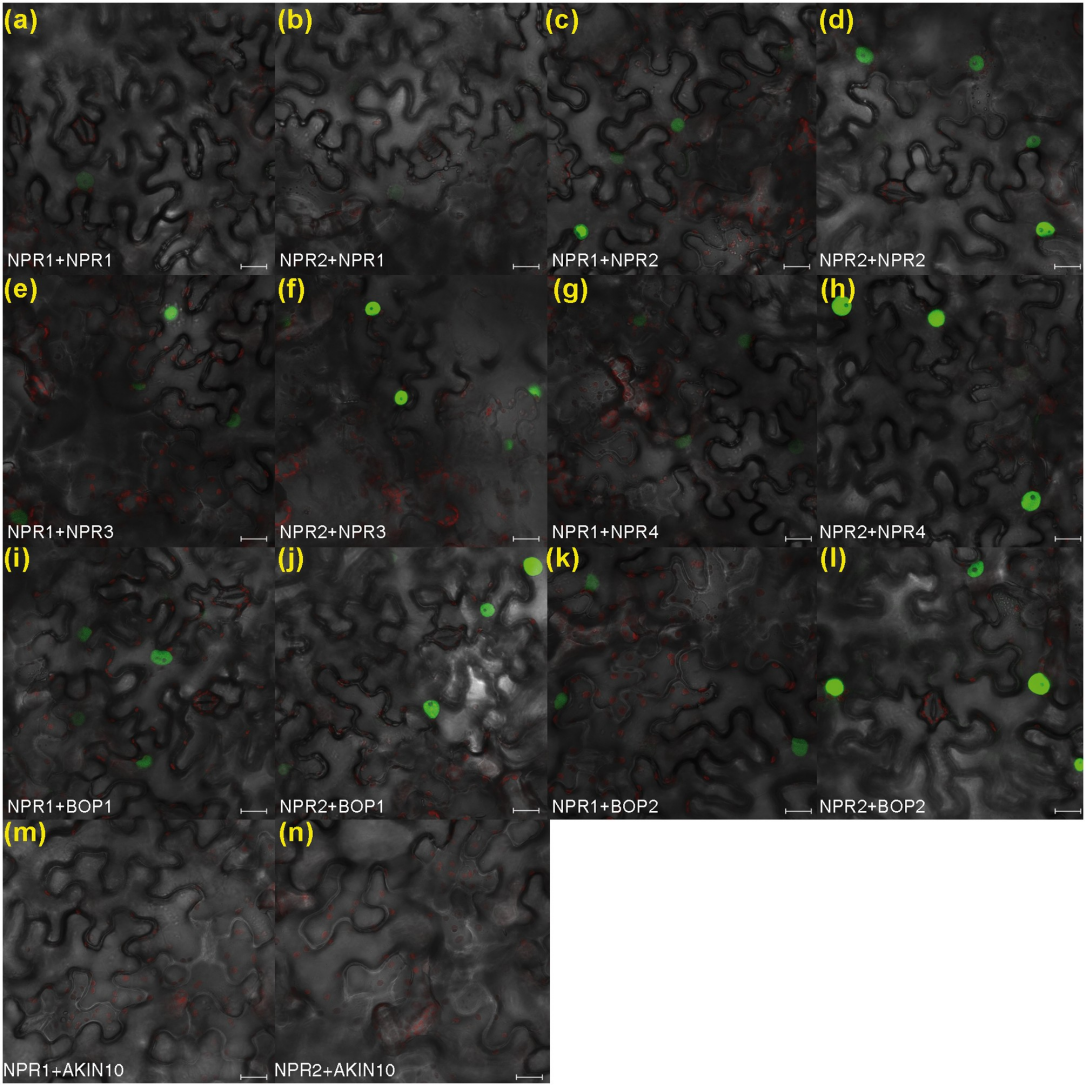
*In planta* interactions among NPR1 paralogs. Bimolecular fluorescence complementation (BiFC) among NPR1 paralogs. The constructs were agroinfiltrated with 1mM SA treatment one day before visualization. The first protein is fused with the N-terminal part of GFP, and the second with the C-terminal. (a) NPR1 and NPR1. (b) NPR2 and NPR1. (c) NPR1 and NPR2. (d) NPR2 and NPR2. (e) NPR1 and NPR3. (f) NPR2 and NPR3. (g) NPR1 and NPR4. (h) NPR2 and NPR4. (i) NPR1 and BOP1. (j) NPR2 and BOP1. (k) NPR1 and BOP2. (l) NPR2 and BOP2. (m) NPR1 and AKIN10, as a negative control[37]. (n) NPR2 and AKIN10, as a negative control. The bars in these pictures represent 20 μm.

After the yeast, we tested the interactions among the NPRs in *N*. *benthamiana* with transient expression. We checked by Bimolecular Fluorescence Complementation (BiFC [49]), and Fig 6 shows the results of the interactions between NPR1 or NPR2 with the NPRs. In general, we detected interactions in all the cases, with NPR2 giving a stronger signal than NPR1.

**Fig 7:**
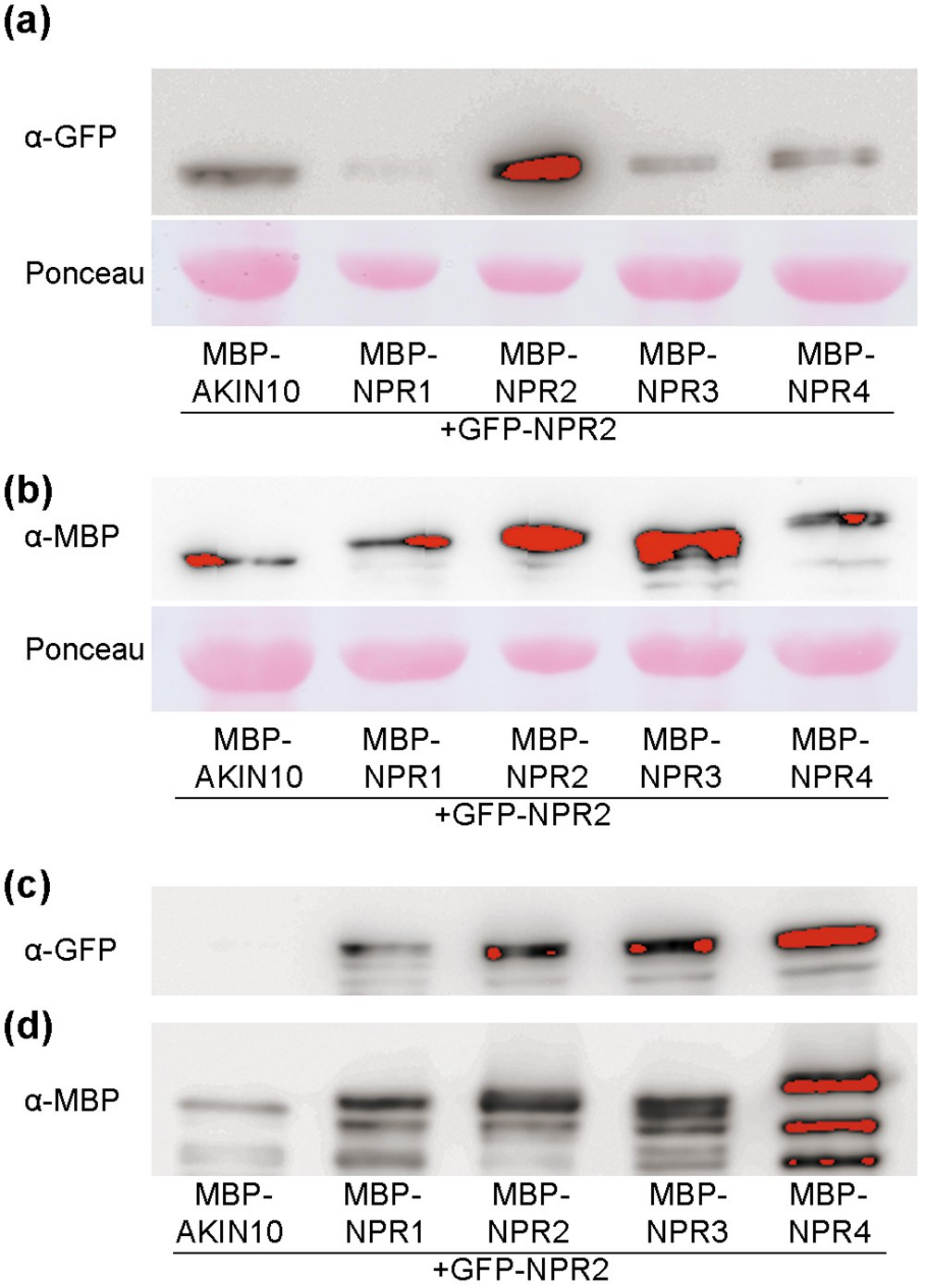
Co-sedimentation among paralogs. (a) GFP-NPR2 and the MBP fused to some NPR1 paralogs were transiently expressed in *N*. *benthamiana* by agroinfiltration and pulled-down with amylose resin. MBP-AKIN10 [37] is an unrelated protein, used as negative control with a similar size to the MBP-NPRs. The panel shows GFP-NPR2 detected by immunoblot before treatment with resin. Ponceau-S staining of the nitrocellulose membrane is shown as a loading control. (b) Expression of MBP-NPRs detected by immunoblot analysis before treatment with resin. (c) The panel shows the eluted fraction from the resin detecting GFP-NPR2 by immunoblot analysis. (d) The same eluted fraction detecting MBP-NPRs.

A better way to determine interactions between two proteins is to coprecipitate them. Fig 7 shows such an experiment. We transiently expressed *GFP-NPR2* in *N*. *benthamiana* (Fig 7a) and *MBP* fused to several *NPRs* (Fig 7b). After the NPRs were pulled down by affinity, NPR2 was identified in the precipitate. In these conditions, NPR2 interacts with itself, NPR1, NPR3, and with NPR4 (Fig 7c). Fig 7d shows the purified MBP-NPRs as controls. We used MBP-AKIN10 [37], an unrelated protein, as a control. Note that the amount of MBP-AKIN10 recovered is less than the MBP-NPRs, so that this control needs to be taken with some caution.

### Protein stability and function of the NPRs

**Fig 8:**
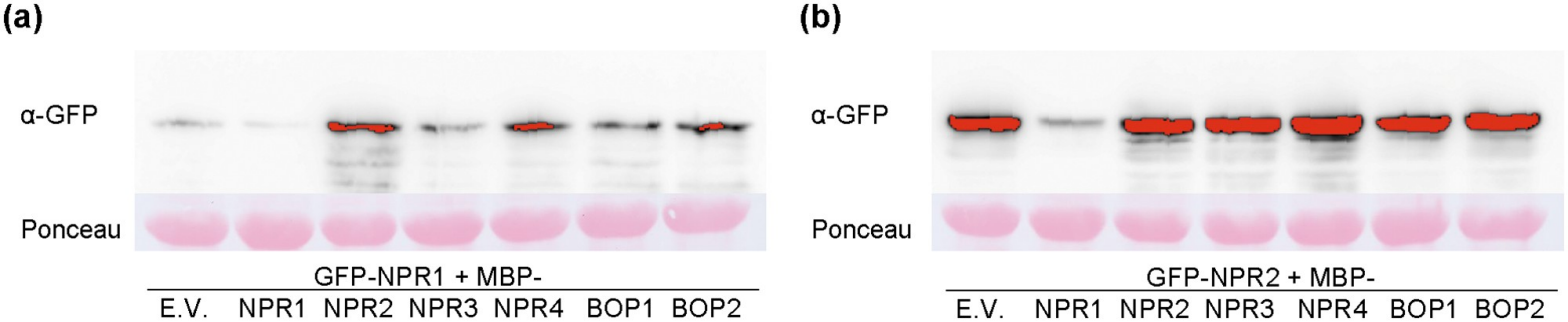
Stability of NPR1 and NPR2 in the presence of the *NPR1* paralogs. (a) Stability of GFP-NPR1 when co-expressed with the NPR1 paralogs fused to MBP. The constructs were agroinfiltrated with 1mM SA treatment one day before sampling. (b) Stability of GFP-NPR2 when co-expressed with the NPR1 paralogs, as in (a). Ponceau-S staining is shown below as a loading control.

NPR3 and NPR4 regulate the stability of NPR1 [19]. We wondered if the same regulation affects NPR2, so we coexpressed NPR1 with the paralogs (Fig 8a) and NPR2 with the paralogs (Fig 8b). With NPR1, we did not see any evidence of this regulation, in fact there is less GFP-NPR1 in the presence of MBP-NPR1 than in the presence of MBP alone (Fig 8a). It is true that in the presence of NPR3, there is less NPR1 than with other paralogs, but the levels of protein are still above the control with MBP alone. In the case of NPR2, the levels of protein are quite constant, except in the presence of MBP-NPR1 (Fig 8b).

**Fig 9:**
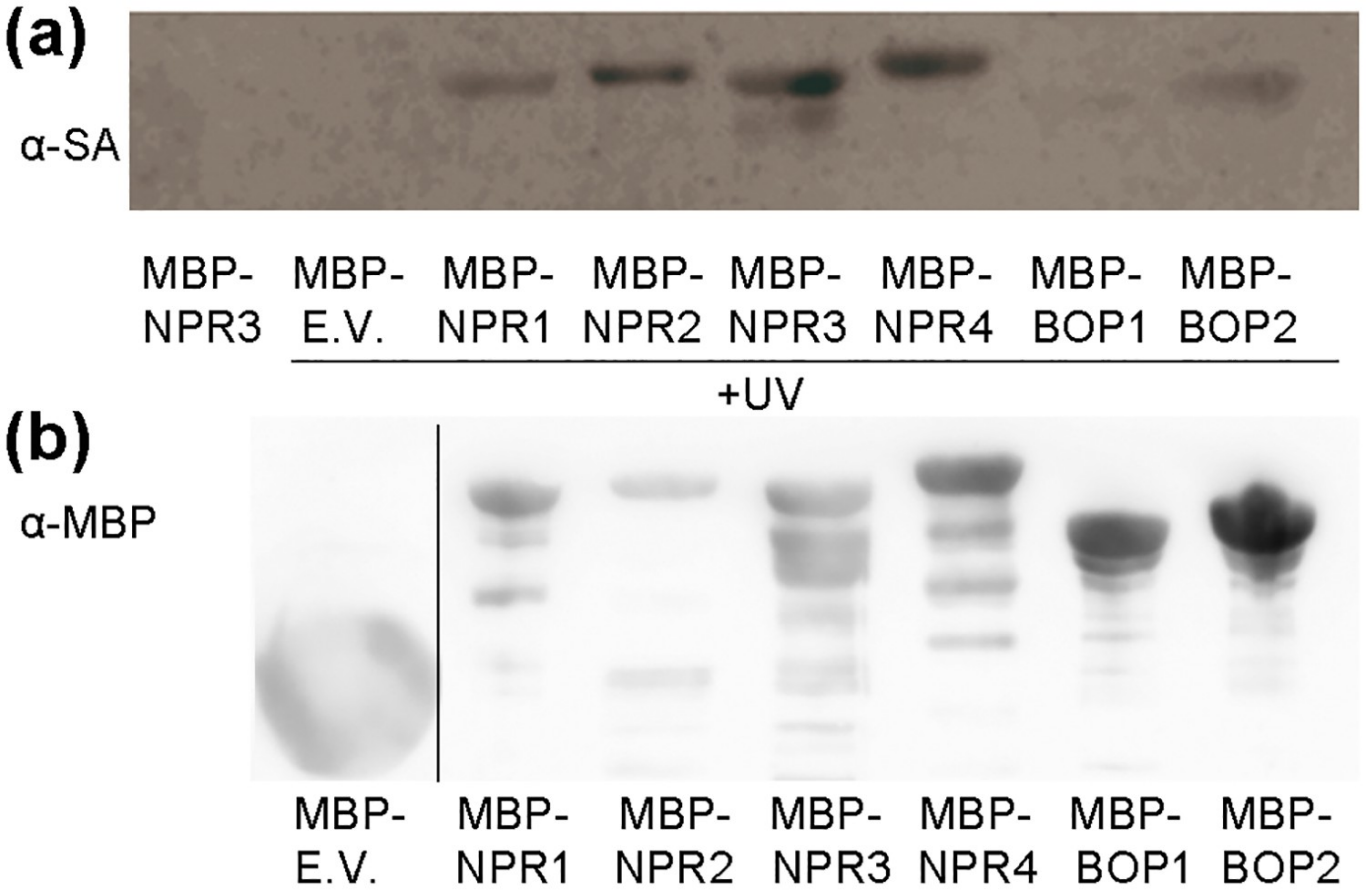
SA binding of the *NPR1* paralogs. (a) Purified recombinant proteins were incubated with 4-AzSA, then treated with UV light. 4-AzSA-cross-linked proteins were detected by immunoblot analysis with anti-SA antibody. The first line corresponds to a negative control, where the UV light was omitted. (b) Loading control, the same amount of protein used in A was detected with an anti-MBP. The image was cut to show the MBP empty vector, as indicated by the vertical line.

NPR1, NPR3, and NPR4 bind SA [15];[19]. Perhaps this characteristic is common to all the NPRs, or perhaps is only found in the NPRs that have a direct function in SA perception. We analyzed the SA binding of all the NPRs together [41], and Fig 9a shows that all the NPRs have some SA binding with this particular technique, while Fig 9b shows the amount of protein used in the experiment. In this assay, BOP1 and BOP2 had a very weak binding, NPR3 and NPR4 had strong activity, and as we would expect given the presented phenotypes, NPR2 bound SA stronger than NPR1.

## Discussion

### NPR2 is part of the SA perception

NPR1 is the master regulator of SA perception, a position reached by genetic (e.g. [7]), biochemical (e.g. [15]), and molecular approaches (e.g. [50]). However, there are several lines of evidence that suggest other genes participate in SA signalling. First, NPR3 and NPR4 bind SA and negatively regulate the levels of NPR1 [19]. Secondly, plants with *npr1* null mutation retain a part of the SA perception [9]. Third, there are genes induced by SA independently of NPR1 [28].

NPR2, in spite of being more similar to NPR1 than the other NPRs, has not been studied in detail until now. We considered the hypothesis that NPR2 could act with NPR3 and NPR4, or alternatively, with NPR1. NPR2 complemented an *npr1-1* mutation (Fig 1), while the other NPRs did not. NPR2 and NPR1 were not interchangeable, since NPR2 was not as efficient as NPR1 in complementing the different phenotypes. Moreover, in our experiments NPR2 was more stable than NPR1, measured as protein accumulation (Fig 8, S1, S2 and S3 Figs).

The phenotypes produced in the *35S*:*NPR2* plants could be attributed to the ectopic and unregulated overexpression, although none of the other NPRs have these phenotypes. However, while the mutant *npr2* did not have a phenotype in our conditions, the presence of *npr2* did produce a phenotype when combined with *npr1-1* or other *nprs* (Fig 2). Therefore, NPR2 plays a role in SA perception.

If NPR2 plays a role in SA perception, it has to be in the right place, meet with the right proteins, and with the right chemical. NPR2 localized in the nucleus and did not change its localization upon SA treatment (S2 and S3 Figs). This behaviour is different from NPR1, which concentrates in the nucleus in the presence of SA [30]. But NPR1 is the exception, since none of the others NPRs were affected by the SA (S2 and S3 Figs). In fact, a detailed study of two NPR1 orthologs from tobacco showed a continuous nuclear localization [26].

Regarding the interactions, NPR2 clearly interacted with NPR1. Note that in our yeast system (see Methods for details), the interaction between NPR2 and NPR1 was the strongest one (Fig 5), with other interactions present with less stringent conditions, including the interaction between NPR4 and NPR2 in the presence of SA (Fig 5). This result partially disagrees with some of the published interactions in yeast [19], although no interactions were found between NPR1 and NPR3/4 in yeast in a recent work [20]. Our data demonstrated that NPR1 interacts with NPR4 in the absence of SA and this interaction is disrupted by SA as published (Fig 5 [19]). We attribute this discrepancy to the use of a different system, which in our case is known to be more restrictive [51]. In fact, the yeast system reproduced the interaction of NPR1 with itself, which can be detected *in planta* [52], only in the less stringent conditions (Fig 5). In the BiFC system all the NPRs interacted with the others (Fig 6), but a more detailed experiment of co-sedimentation showed that NPR2 interacted *in planta* with NPR3 and NPR4 to some degree, as reported for NPR1. Moreover, NPR2 also interacted with NPR1 and with itself (Fig 7). NPR2 also interacted with part of the NPR1 interactors like three TGAs (Fig 4), so the connection with the gene activation upon SA perception is direct.

NPR3/4 have been proposed to be relevant in SA perception by regulating the amount of NPR1 [19], and perhaps NPR2 could regulate the amount of NPR1 in opposition to NPR3/4. The results in transient assays in *N*. *benthamiana* showed that, in the presence of NPR2, there was indeed an increase of NPR1 with respect to the empty vector control (Fig 8). It was not a symmetric interaction, since NPR1 reduced the levels of NPR2, with no effect on the rest of NPRs in these conditions (Fig 8). We did not detect a decrease of NPR1 in the presence of NPR3 or NPR4 as reported [19], perhaps due to the transient nature of our experiment.

NPR2 binds SA at least as strongly as NPR1 (Fig 9). That NPR1 binds SA has been described before [15];[53], and the same is true for NPR3/4 [19]. There was even a small amount of SA binding in BOP1/2 in this assay, requiring further confirmation by other methods. Although there was no apparent phenotype with SA in the *bop1 bop2* mutant [29] (Fig 2), these weak interactions could explain the phenotypes in some combinations of mutants, described below.

To sum up, our results show that *NPR2* can complement *npr1*, the lack of *NPR2* produces a measurable phenotype, NPR2 interacts with some of the proteins with which NPR1 interacts, and binds SA. Therefore NPR2 acts as a SA receptor in the same way as NPR1.

What is the role of NPR2 *in planta*? NPR2 reaches peak expression in senescent leaves and later stages of fruit formation (including dry seeds [54]). In terms of global expression, *NPR2* is expressed five times lower than *NPR1* ([55]). The data of *NPR2* expression could explain why its role in SA perception is only detected when ectopically expressed, or in an *npr1* background. In normal conditions, we speculate that *NPR2* would play a role in SA perception in the mentioned peaks of expression, and also as an evolutive reservoir for NPR1.

### SA dependent, NPRs independent gene expression

Since *npr2* has a phenotype with SA in combination with *npr1-1*, we reasoned that perhaps the genes induced by SA in an *npr1*-1 mutant (*SdNi*) would alter their expression in an *npr1-1 npr2* background. When we performed the experiment, the results were not as clear-cut as expected (Fig 3). In four cases, the sextuple mutant is more extreme than *npr1-1* (either in induction or repression), while in two the sextuple is closer to wt than to *npr1-1*. In the four cases where *npr1*-*1 npr2* was tested alongside the sextuple, there was no significant difference between *npr1-1* and *npr1-1 npr2*. Thus, instead of some genes being *NPR1* and *NPR2* dependent or independent, it would be more accurate to describe them as *NPRs* dependent or independent. It is clear that part of the *SdNi* genes are *NPRs* dependent. In the case of the gene *GRX480*, which we could call *NPRs* independent in the short term, it is more difficult to categorize in the long term (Fig 3).

The expression of *SdNi* genes did not have an impact on the defence or growth of the plant with SA that we could measure, since *npr1-1* plants with SA are as susceptible as with a mock treatment (Fig 2c). Perhaps this gene activation is unrelated to the defence response and is a consequence of the chemical characteristics of SA. In the four genes tested, HBA did not have the same effect as SA, so it seems that this gene activation is SA specific. It is possible that there are additional receptors for SA besides the NPRs, and perhaps these putative receptors are relevant for the set of responses to SA in development [56].

### The rest of the paralogs regulate SA perception

*BOP1/2* are two paralogs of *NPR1* with a defined role in development [29]. We found that the rest of paralogs did not have a function in the same phenotypes affected by *BOP1/2* (S5 Fig), so their role in development is not shared among the *NPRs*. Since the sextuple is viable, we can say that there is no other obvious function in development shared by the *NPRs*. The defensive role of *BOP1/2* was considered to affect only part of the resistance triggered by MeJA [23], but the work here presented shows that they also affect SA response (Fig 2a and 2b). Since *npr3 npr4 bop1 bop2* plants respond more strongly to BTH than their parentals *npr3 npr4*, and *bop1 bop2*, perhaps *BOP1/2* have the same function as that attributed to *NPR3/4*, namely to regulate the levels of NPR1 [19]. The effect of the *bops* is only perceived when they are in an *npr3 npr4* background, so there is a hierarchy, where *NPR3/4* are more relevant, and *BOP1/2* are less relevant for this phenotype. The effect of the BOPs could be through their interaction with NPR2 and NPR4 (Fig 5), or perhaps due to competition for shared factors between BOP1/2 and the rest of the paralogs. It has been said that BOP1/2 have a different C-terminal domain than NPR1 and NPR3/4, a domain involved in SA-binding in these paralogs [15]; [20]. The response of these genotypes in the growth altered by BTH could be considered unique, since it was not observable in the growth of the pathogen (Fig 2c). However, Fig 3 shows that in three out of four genes tested, there was a stronger induction by SA in *npr3 npr4 bop1 bop2* with respect to the wt. Therefore, this is not a unique phenotype of BTH, or related to growth, but related to SA perception, and NPR3/4 and BOP1/2 negatively regulate SA perception.

At the same time, the sextuple mutant pointed in the opposite direction, where not only NPR2, but all the NPRs positively participate in SA perception. The best example was *PR1* (Fig 3c). While in an *npr1-1* background the levels of *PR1* were quite low (giving NPR1 its name), in the sextuple mutant the levels were even lower. In the rest of the genes and conditions, the sextuple mutant behaved differently than *npr1-1* in all but in *GRX480* at short times. Note that in some cases this difference took the sextuple closer to wt than to *npr1-1* (Fig 3a and 3b). Thus, besides NPR1, some of the SA signalling goes through the NPRs, and yet part of the SA signalling is going through unknown mechanism(s).

This contradiction between a positive and a negative role for *NPR3/4* and *BOP1/2* could be explained by the difference in their background. The evidence for a positive role is presented in an *npr1-1* background, while the negative role is presented in an *NPR1* background. Consequently the difference in behaviour is due to the relation of NPR3/4 and BOP1/2 with NPR1. Since these four proteins bind SA to some degree, we suggest that although these proteins perceive SA, they are less efficient in producing the signal. When NPR1 is present, these four proteins bind SA and act as competitors to NPR1. When NPR1 and NPR2 are not functional, NPR3/4 and BOP1/2 (in small amounts) are able to trigger SA signalling.

## Supporting information

S1 Fig Several *NPRs* phenocopy *NPR1* in *Nicotiana benthamiana*. (PDF)

S2 Fig Expression and localization of the NPR1 paralogs. (PDF)

S3 Fig Expression and localization of NPR2 in Arabidopsis. (PDF)

S4 Fig Behaviour of the mutants in the *NPR1* paralogs in SA plates. (PDF)

S5 Fig Macroscopic phenotype of *npr1-1 npr2 npr3 npr4 bop1 bop2*. (PDF)

S6 Fig Triple interactions in yeast. (PDF)

S1 Table Primers used in this work. (PDF)
