# Supplementary material for: NPR1 paralogs of Arabidopsis and their role in salicylic acid perception
Source: PLoS One. 2018 Dec 28;13(12):e0209835. doi: 10.1371/journal.pone.0209835 (PMC6310259; doi:10.1371/journal.pone.0209835)
Supplement: S1 Fig — (PDF) [file pone.0209835.s001.pdf]

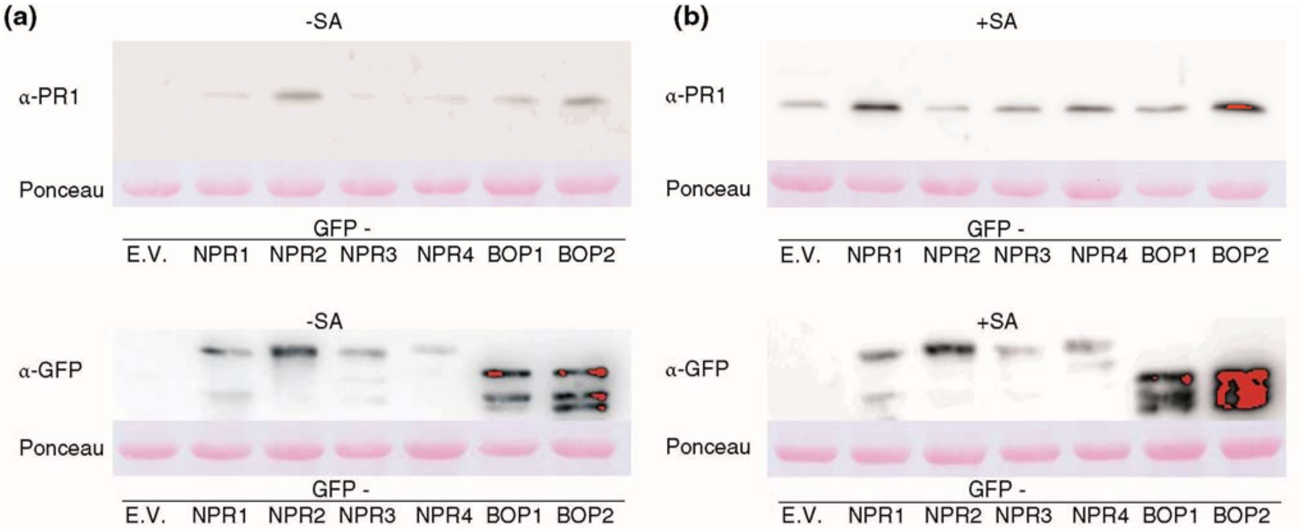

S1 Fig -Several *NPRs* phenocopy *NPR1* in *Nicotiana benthamiana*. Different constructions of GFP fused with the paralogs were transiently expressed in *N. benthamiana* by agroinfiltration. Two days later, either a (a) mock or a (b) 1 mM salicylic acid (SA) treatment was applied, and after one additional day the levels of PR1 were detected by immunoblot, as a measurement of SA perception. The same extracts were used to detect the paralogs with anti-GFP. In each immunoblot, the membrane is shown after staining with Ponceau as a loading control.
