# Supplementary material for: NPR1 paralogs of Arabidopsis and their role in salicylic acid perception
Source: PLoS One. 2018 Dec 28;13(12):e0209835. doi: 10.1371/journal.pone.0209835 (PMC6310259; doi:10.1371/journal.pone.0209835)
Supplement: S2 Fig — (PDF) [file pone.0209835.s002.pdf]

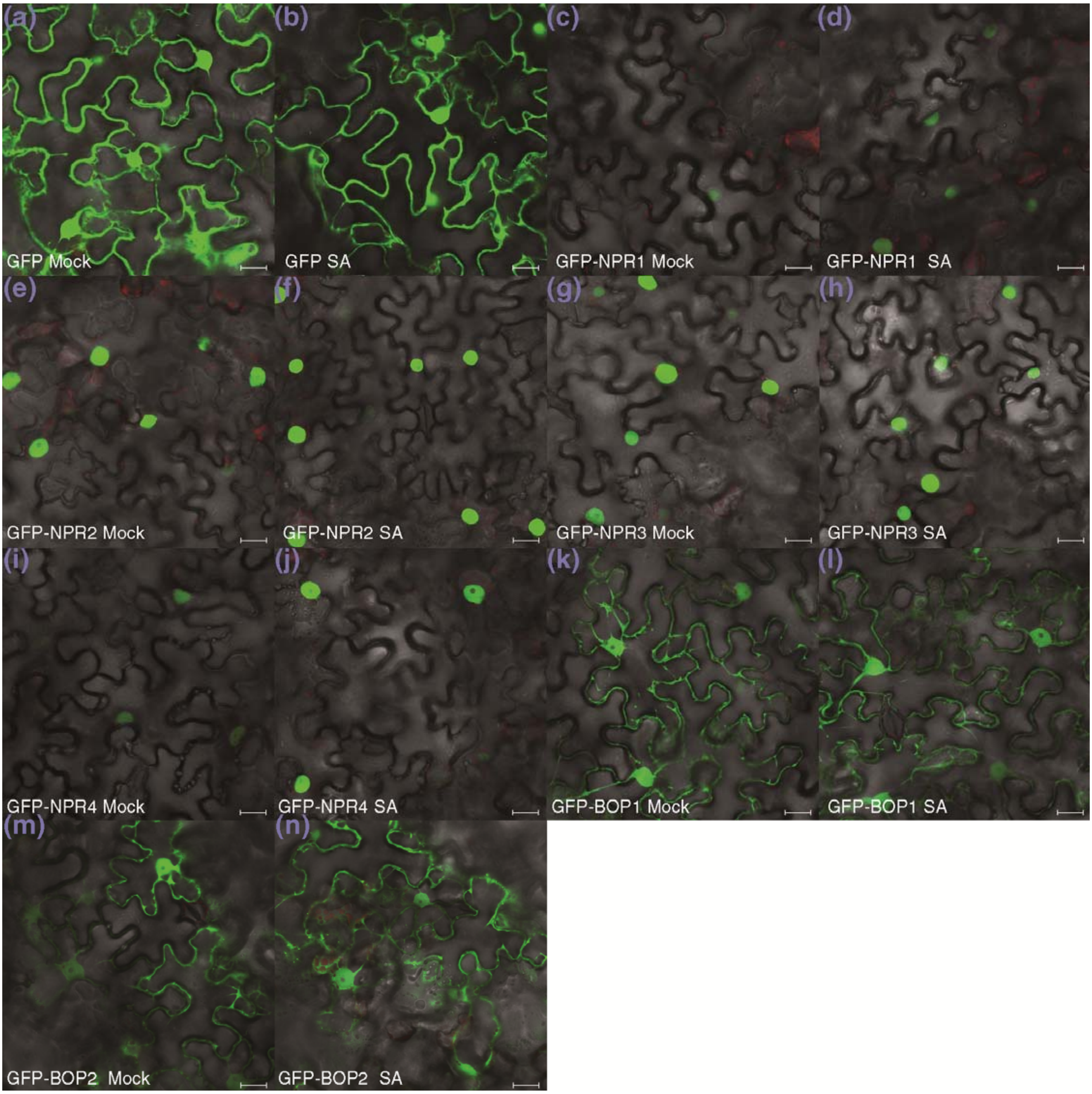

S2 Fig -Expression and localization of the NPR1 paralogs. Transient expression of the NPR1 paralogs in *N. benthamiana* fused with GFP and observed with the confocal microscope. *35S:GFP* with a (a) mock and (b) SA treatment. *35S:GFP-NPR1* with a (c) mock and (d) SA

treatment. *35S:GFP-NPR2* with a (e) mock and (f) SA treatment. *35S:GFP-NPR3* with a (g) mock and (h) SA treatment. *35S:GFP-NPR4* with a (i) mock, and (j) SA treatment. *35S:GFP-BOP1* with a (k) mock, and a (l) SA treatment. *35S:GFP-BOP2* with a (m) mock and (n) SA treatment. The bars in these pictures represent 20 µm.
