# Supplementary material for: NPR1 paralogs of Arabidopsis and their role in salicylic acid perception
Source: PLoS One. 2018 Dec 28;13(12):e0209835. doi: 10.1371/journal.pone.0209835 (PMC6310259; doi:10.1371/journal.pone.0209835)
Supplement: S3 Fig — (PDF) [file pone.0209835.s003.pdf]

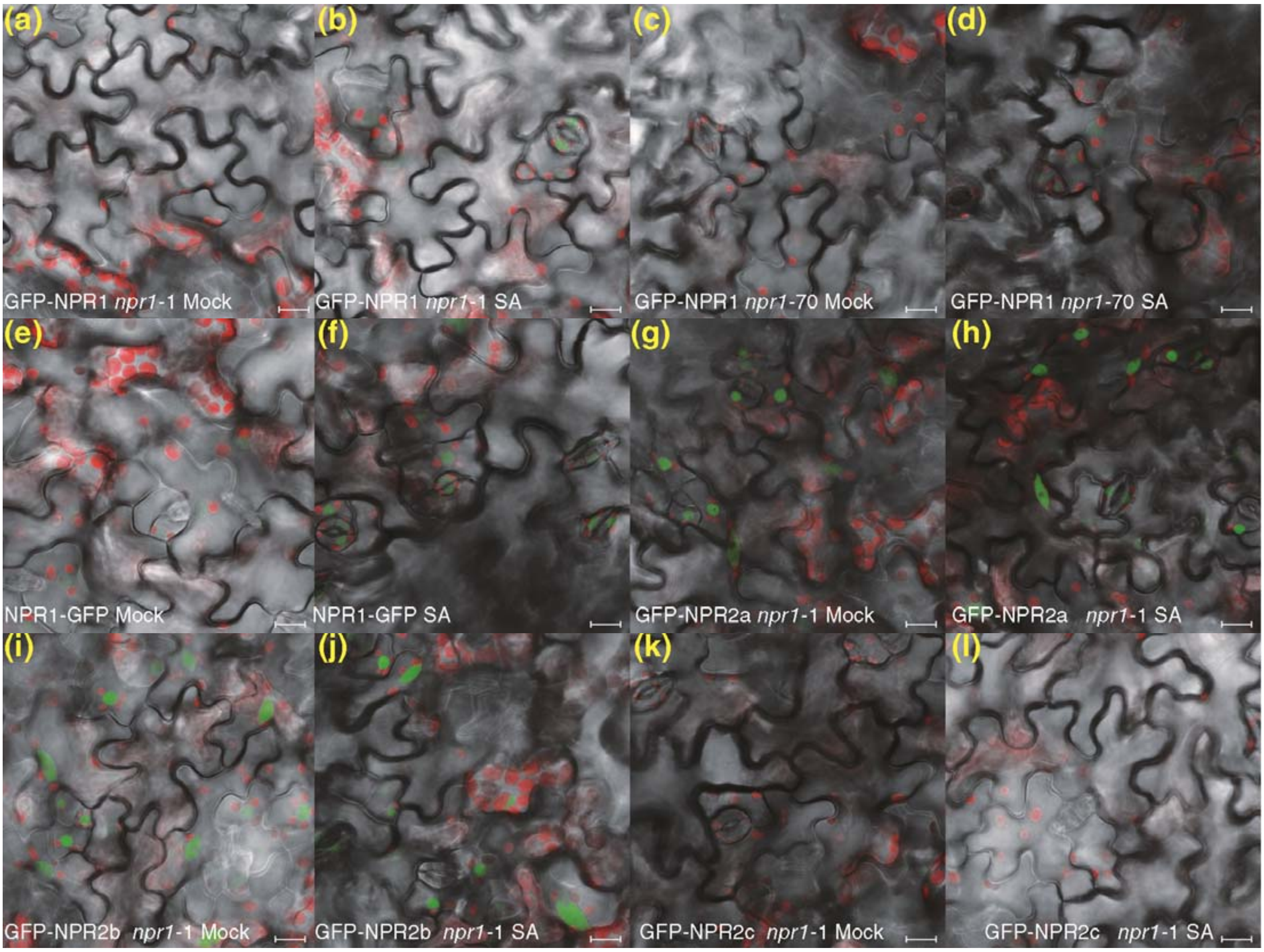

S3 Fig -Expression and localization of NPR2 in Arabidopsis. Homozygous transgenics expressing GFP fused to NPR1 or NPR2 were observed with the confocal microscope. *35S:GFP-NPR1* in *npr1*-1 background after (a) mock and (b) SA treatment. *35S:GFP-NPR1* in *npr1*-70 background after (c) mock and (d) SA treatment. *35S:NPR1-GFP* in wild type background after (e) mock treatment and (f) SA treatment. *35S:GFP-NPR2a* in *npr1*-1 background after (g) mock and (h) SA treatment. *35S:GFP-NPR2b* in *npr1*-1 background after (i) mock and (j) SA treatment. *35S:GFP-NPR2c* in *npr1*-1 background after (k) mock and (l) SA treatment. The bars in these pictures represent 10 µm.
