# Supplementary material for: NPR1 paralogs of Arabidopsis and their role in salicylic acid perception
Source: PLoS One. 2018 Dec 28;13(12):e0209835. doi: 10.1371/journal.pone.0209835 (PMC6310259; doi:10.1371/journal.pone.0209835)
Supplement: S4 Fig — (PDF) [file pone.0209835.s004.pdf]

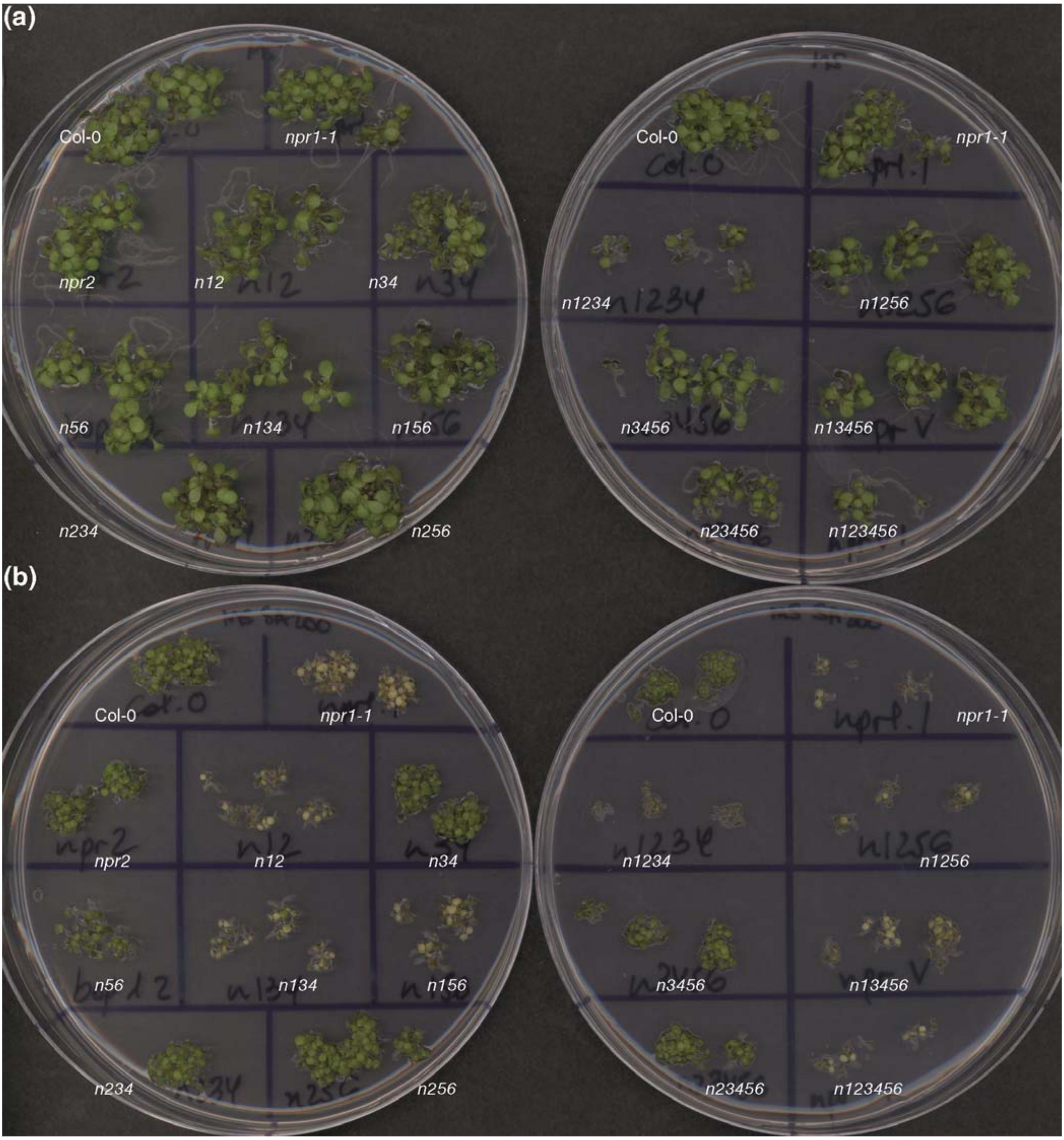

S4 Fig -Behaviour of the mutants in the *NPR1* paralogs in SA plates. (a) As in Fig 1, plants of the indicated genotypes were grown in MS plates. (b) The same phenotypes with 200 µM SA. The presence of *npr1*-1 is essential for the bleaching phenotype in SA, and the absence of the *NPR1* paralogs does not make any difference.
