# Supplementary material for: NPR1 paralogs of Arabidopsis and their role in salicylic acid perception
Source: PLoS One. 2018 Dec 28;13(12):e0209835. doi: 10.1371/journal.pone.0209835 (PMC6310259; doi:10.1371/journal.pone.0209835)
Supplement: S5 Fig — (PDF) [file pone.0209835.s005.pdf]

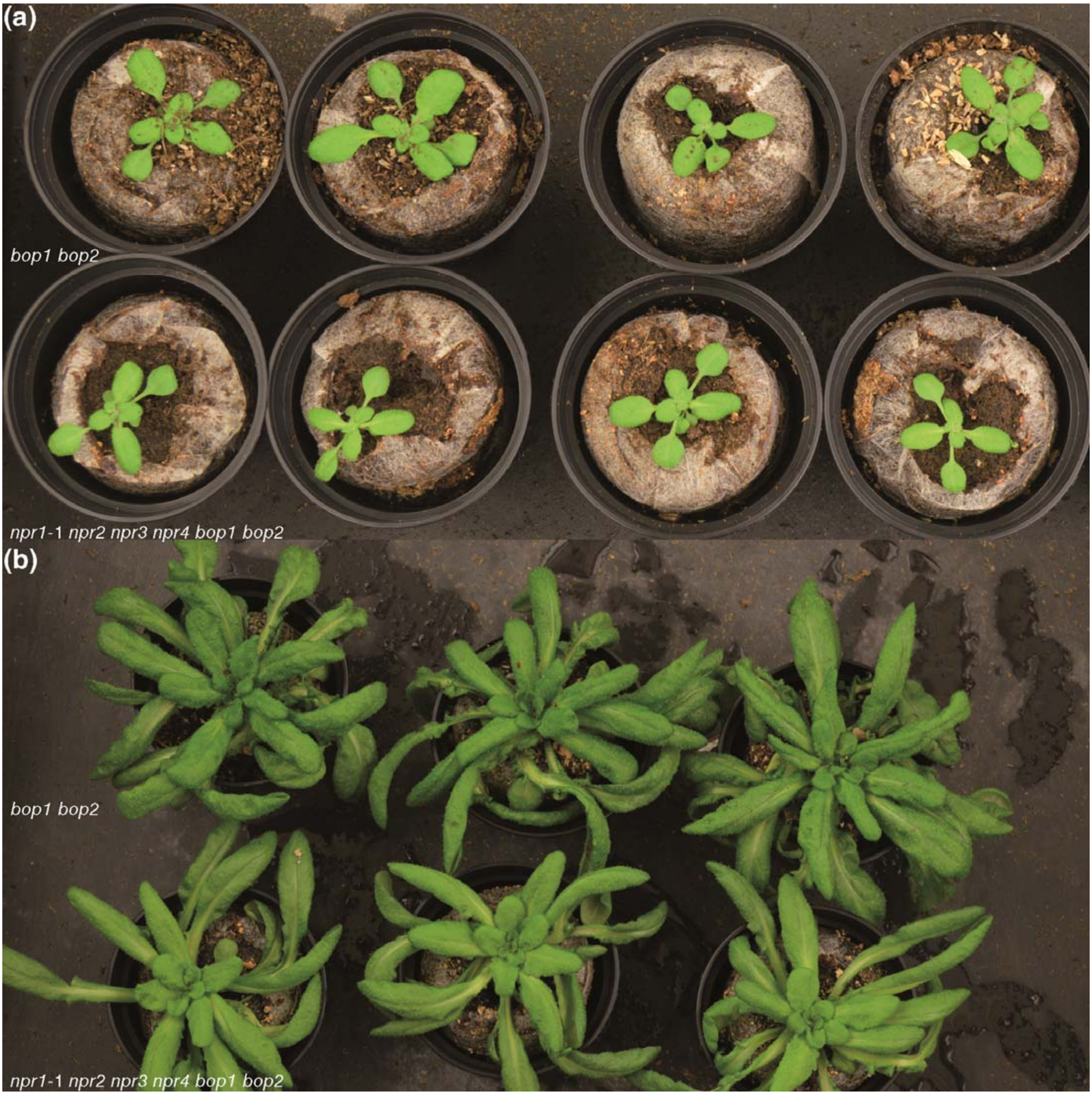

S5 Fig -Macroscopic phenotype of *npr1*-1 *npr2 npr3 npr4 bop1 bop2*. Plants *bop1 bop2* and the sextuple *npr1-1 npr2 npr3 npr4 bop1 bop2* were grown in the same conditions (short day) to observe any changes in the blade-on-petiole phenotype. (a) Picture taken at four weeks. (b) Picture taken at eight weeks. We did not observe any difference in phenotype macroscopically.
