# Supplementary material for: NPR1 paralogs of Arabidopsis and their role in salicylic acid perception
Source: PLoS One. 2018 Dec 28;13(12):e0209835. doi: 10.1371/journal.pone.0209835 (PMC6310259; doi:10.1371/journal.pone.0209835)
Supplement: S6 Fig — (PDF) [file pone.0209835.s006.pdf]

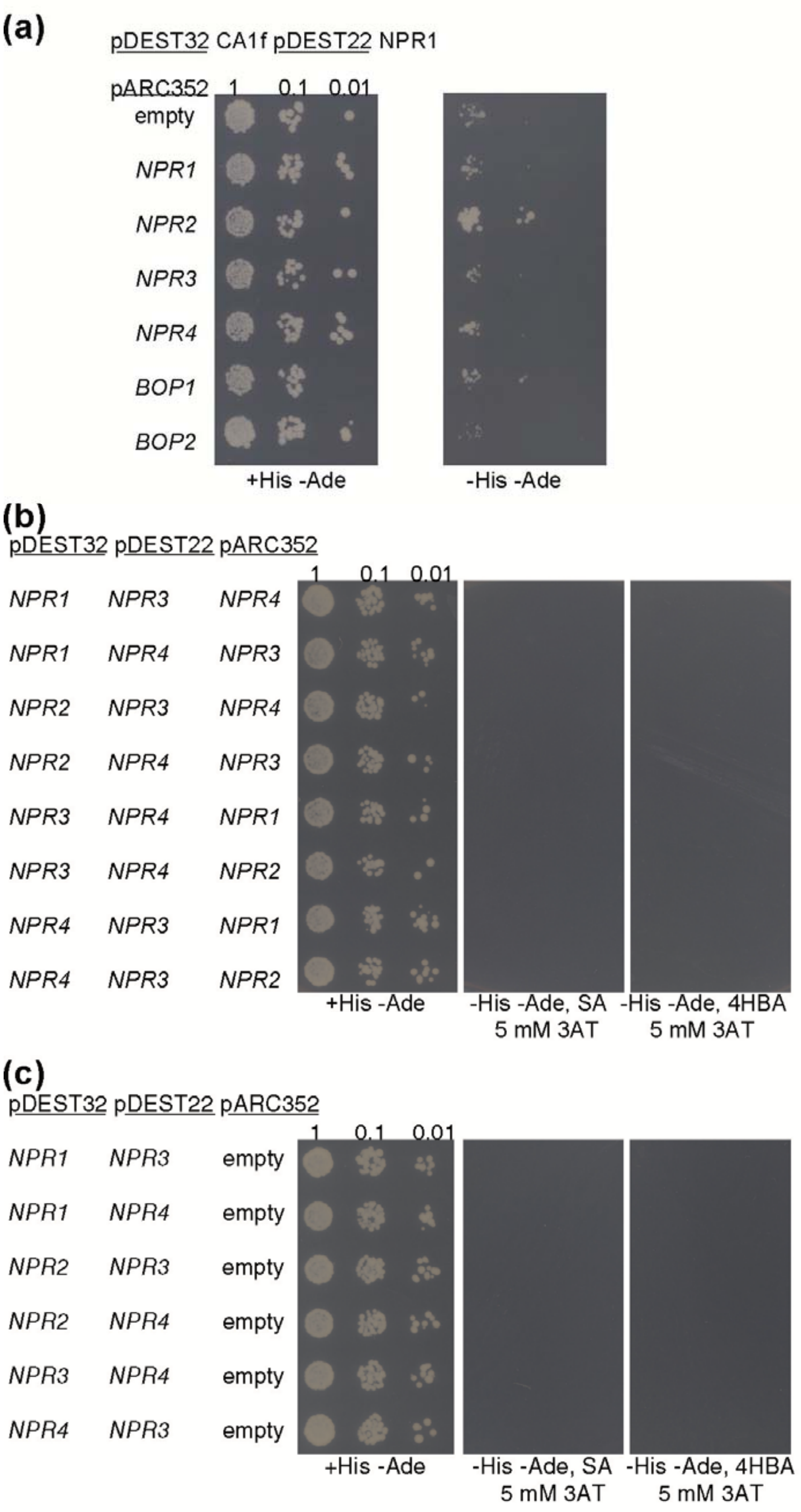
(a)
pDEST32 CA1f pDEST22 NPR1
pARC352 1 0.1 0.01
empty
NPR1
NPR2
NPR3
NPR4
BOP1
BOP2
+His -Ade
-His -Ade
(b)
pDEST32 pDEST22 pARC352
1 0.1 0.01
NPR1 NPR3 NPR4
NPR1 NPR4 NPR3
NPR2 NPR3 NPR4
NPR2 NPR4 NPR3
NPR3 NPR4 NPR1
NPR3 NPR4 NPR2
NPR4 NPR3 NPR1
NPR4 NPR3 NPR2
+His -Ade
-His -Ade, SA 5 mM 3AT
-His -Ade, 4HBA 5 mM 3AT
(c)
pDEST32 pDEST22 pARC352
1 0.1 0.01
NPR1 NPR3 empty
NPR1 NPR4 empty
NPR2 NPR3 empty
NPR2 NPR4 empty
NPR3 NPR4 empty
NPR4 NPR3 empty
+His -Ade
-His -Ade, SA 5 mM 3AT
-His -Ade, 4HBA 5 mM 3AT

S6 Fig -Triple interactions in yeast. (a) Triple interactions between NPR1, βCA1f and the paralogs. In this case, the plates are +His-Ade and -His-Ade, since NPR1- βCA1f interacts in the presence of SA. (b) Triple interactions among the paralogs. The plates are -Ade +His, -Ade -His +5 mM 3AT+100 µM SA, and -Ade-His+5 mM 3AT+100 µM HBA. (c) Controls of the background of (b). The numbers indicate the concentration of yeast, as OD600.
