# Supplementary material for: NPR1 paralogs of Arabidopsis and their role in salicylic acid perception
Source: PLoS One. 2018 Dec 28;13(12):e0209835. doi: 10.1371/journal.pone.0209835 (PMC6310259; doi:10.1371/journal.pone.0209835)
Supplement: S1 Table — (PDF) [file pone.0209835.s007.pdf]

S1 Table -Primers used in this work.

Name	Sequence	Use
TP492	TGTTTGGTAAATTGGAGACATGCGAAGAGG	For npr2 marker
TP493	ACCGCCACAACCGAGTCAAAGCC	For npr2 marker
TP501	CAAACCAGCAATGTCAAGGTACCAT	For npr4 marker (Forward)
TP502	AACGCAGTAGAGGAAAACACAATTCAA	For npr4 marker (Reverse mut)
TP504	TGCACCTGTTGTGTCCTGTTTGT	For npr3 marker (Forward)
TP505	TGCCAATTAATGCCGTCACG	For npr3 marker (Reverse mut)
TP506	CGAAACCTCCAATGGAAAAGGC	For npr3 marker (Reverse wt)
TP507	ATTAGCATTAGGTGACGCAGACAC	For bop1 marker
TP508	GATTAATGTCGACGTTTGTGCTTT	For bop1 marker
TP509	CTTAAGCTTTTTCCCCCGGAAA	For bop2 marker
TP510	CATCCTCTCTCGCCACAATTAGGT	For bop2 marker
TP514	GGTGTTTCGTAACTCGCCTCTGT	For npr4 marker (Reverse wt)
TP672	GGGGACAAGTTTGTACAAAAAAGCAGGCTTCATGGCCACCACCACCACCACCAC	For cloning NPR2 cDNA
TP673	GGGGACCACTTTGTACAAGAAAGCTGGGTTTTAATCCCCGTTCCCGTAAGGTCG	For cloning NPR2 cDNA
TP674	GGGGACAAGTTTGTACAAAAAAGCAGGCTTCATGGCTACTTTGACTGAGCCATC	For cloning NPR3 cDNA
TP675	GGGGACCACTTTGTACAAGAAAGCTGGGTTTCATGTTGTGTTGTGCAGGTCATC	For cloning NPR3 cDNA
TP676	GGGGACAAGTTTGTACAAAAAAGCAGGCTTCATGGCTGCAACTGCAATAGAGCC	For cloning NPR4 cDNA
TP677	GGGGACCACTTTGTACAAGAAAGCTGGGTTTCATGTTGGATTCTCTAAGGCTTC	For cloning NPR4 cDNA
TP852	CGACTCCAGGAGGTTTTCTCATCAC	qRT-PCR probes for At1g76680
TP853	ACCACCTTTGGCATGTACAGCATCA	qRT-PCR probes for At1g76680
TP854	GGCTCGCAAAATATCCGTACACCTT	qRT-PCR probes for At3g01420
TP855	ACACCAGCCCAACTGTTACGAATG	qRT-PCR probes for At3g01420
TP856	AAAATGAAGGCGGAGTTGAATTTGC	qRT-PCR probes for At5g63790
TP857	CCGATCGGTTTATCAGCTCCAGTC	qRT-PCR probes for At5g63790
TP858	TTAAGGAAGTTGGGGCTCTG	qRT-PCR probes for At1g28480
TP859	CCGTAAACAACAATTACCAATCA	qRT-PCR probes for At1g28480
TP860	GTGGGTTAGCGAGAAGGCTA	qRT-PCR probes for At2g14610
TP861	ACTTTGGCACATCCGAGTCT	qRT-PCR probes for At2g14610

TP862	CCGACGAACGCGAAGCTACT	qRT-PCR probes for At1g05560
TP863	TCTCTACCCGCTTCCATCGCT	qRT-PCR probes for At1g05560
